# Hypertransaminasemia and fatal lung disease: a case report

**DOI:** 10.1186/1824-7288-39-9

**Published:** 2013-02-07

**Authors:** Francesca Santamaria, Sara De Stefano, Silvia Montella, Marco Maglione, Roberto Della Casa, Emma Acampora, Claudio Pignata, Mariacarolina Salerno, Giancarlo Parenti

**Affiliations:** 1 Department of Pediatrics, Federico II University, Naples, Italy

**Keywords:** Pompe disease, Cardiomegaly, Atelectasis, Respiratory failure

## Abstract

Glycogenosis type II (Pompe disease) is a rare autosomal recessive genetic disorder caused by mutations in the gene encoding the lysosomal enzyme acid α-glucosidase. The classic form is characterized by severe cardiac involvement, generalized hypotonia and exitus early in life. Presenting symptoms and signs of the disease may be neglected or underestimated, thus delaying the diagnosis. Respiratory manifestations mainly occur because of respiratory muscle weakness. However, additional mechanisms can favor the development of pulmonary complications that result in fatal respiratory failure. We herein describe a case of an infant with glycogenosis type II presenting with hepatomegaly and hypertransaminasemia, who rapidly developed fatal lung disease.

## Background

Glycogenosis type II, a rare autosomal recessive lysosomal storage disorder (LSD), is caused by mutations in the acid α-glucosidase (GAA) gene encoding for the lysosomal hydrolase GAA that is involved in the breakdown of glycogen into glucose [1].

Although the deficiency of GAA is generalized, muscles appear to be particularly vulnerable to glycogen storage. The clinical manifestations of the disease are thus mainly related to the involvement of skeletal muscles and heart, and are characterized by progressive muscle hypotonia, motor impairment, respiratory failure, and, exclusively in the classic infantile variant, severe hypertrophic cardiomyopathy [1]. The phenotypic spectrum of the disease is broad and ranges from early-onset severe phenotypes to late-onset attenuated clinical forms [1]. The infantile form (Pompe disease) has a rapidly progressive course and exitus occurs usually within two years of age [2]. Infants with Pompe disease present in the first few months of life with feeding problems, poor weight gain, respiratory difficulties frequently complicated by pulmonary infections, or delayed motor milestones. Laboratory findings include elevated levels of serum creatine kinase (CK), aspartate aminotransferase (AST), and lactate dehydrogenase (LDH) [2].

Because most LSD are systemic, virtually all organs may be involved to a greater or lesser degree. Several LSD have airways disease as part of the clinical picture, yet at presentation or as late-onset features [3-6].

Here we report the case of a 6 months old infant with Pompe disease who initially was referred to us for a suspect of liver disease, and rapidly developed a fatal pulmonary complication.

## Case presentation

The child was a full-term girl, born to unrelated healthy parents after an uneventful pregnancy. Parents reported regular growth and denied any clinical problems during the first months of life. In September 2002, at 6 months of age, she was referred to our Department for further diagnostic work-up because of overt hepatomegaly and occasional finding of hypertransaminasemia at routine blood analysis requested for intercurrent upper airways infection. At admission weight and length were appropriate for the age (25th and 50th percentile, respectively). On physical examination macroglossia and severe generalized muscle hypotonia were evident. She had no evidence of jaundice. The heart rate was 150 beats per minute with gallop rhythm and no added murmurs. Peripheral pulses were normal. Blood pressure was 100/70 mmHg. Respiratory rate was 30 breaths per minute, and the transcutaneous oxygen saturation (SaO_2_) at room air was 98%. Lung auscultation revealed good transmission of air without additional pathological sounds. The abdomen was not distended. The liver edge was palpable 4 cm below the costal margin with smooth surface and increased consistency. There was no splenomegaly.

**Figure 1:**
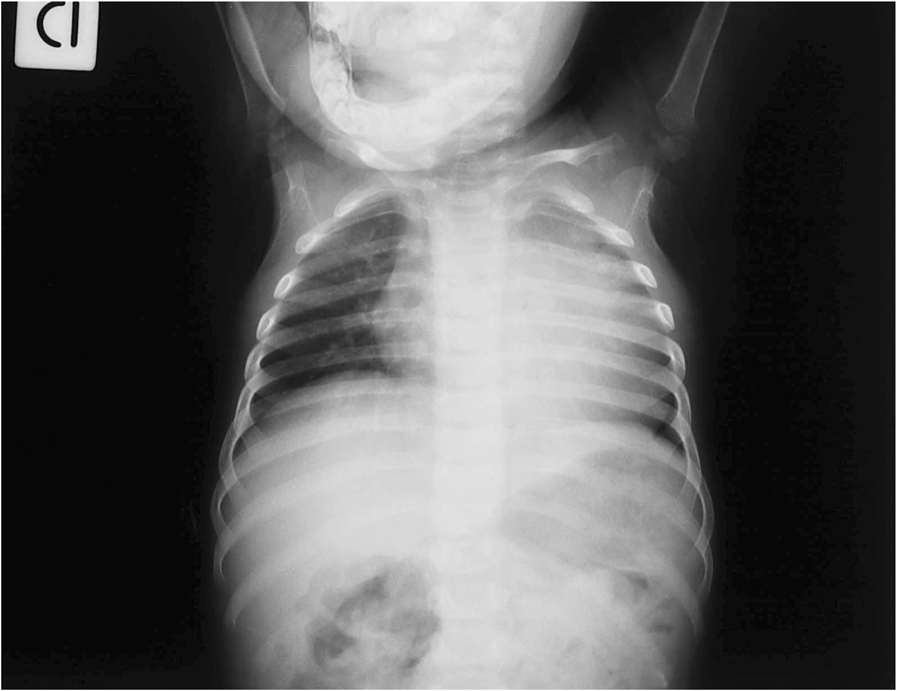
Chest x-ray showing widespread lung hyperinflation and severe cardiomegaly.

The patient underwent blood routine biochemistry and complete cell count. Laboratory findings were all unremarkable, with the exception of hypertransaminasemia [AST = 444 U·l^-1^, alanine aminotransferase (ALT) = 315 U·l^-1^], increased levels of alkaline phosphatase (1428 U·l^-1^), LDH (3925 U·l^-1^), CK (1373 U·l^-1^) and aldolase (26.6 U·l^-1^). Hepatobiliary ultrasound showed a liver of larger size for the age, with no evidence of focal lesions. At chest x-ray diffuse hyperinflation in the absence of any parenchymal consolidation, and cardiomegaly were evident (Figure 1). The electrocardiogram showed a short P-R interval, with signs of left ventricular pressure-overload. The echocardiography revealed the dilation of the left ventricular and atrial cavity, with thickening of the septum and of the walls of the left ventricle, which showed diffuse and severe hypokinesia. Therefore, treatment with digitalis, diuretics and vasodilators was started.

The clinical presentation (cardiomegaly, severe generalized muscular hypotonia, hepatomegaly), and the increase in serum enzymes levels (AST, ALT, CK, aldolase, LDH) raised the diagnostic suspicion towards glycogen storage disease type II. The reduction in acid α-glucosidase activity, measured in cultured fibroblasts (1.6 nmol/mg/h, normal values 64.4 ± 22.9), and the molecular analysis of the GAA gene that showed a homozygous mutation (C1124G> T/C1124G> T) confirmed the diagnosis of Pompe disease.

**Figure 2:**
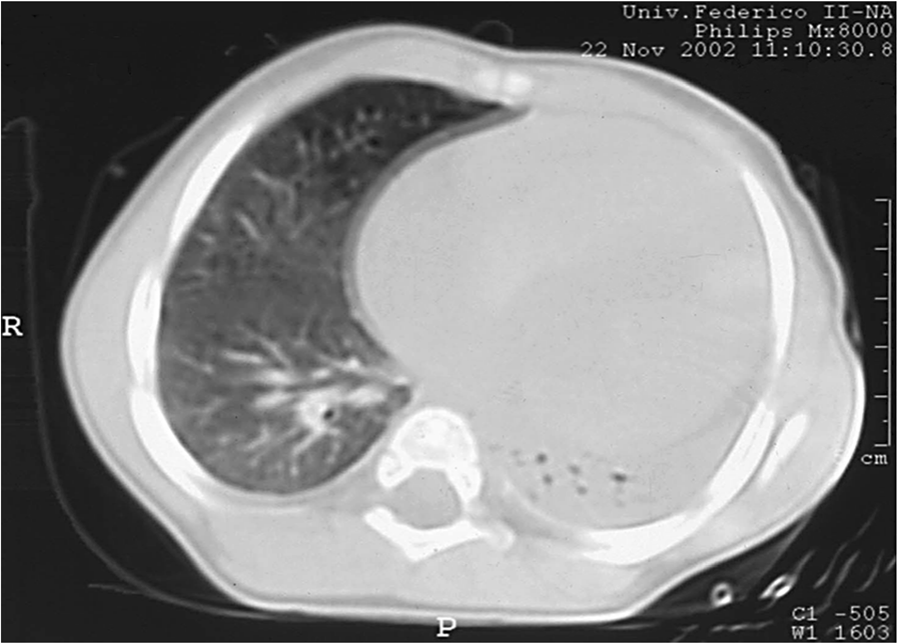
High-resolution computed tomography of the lung, showing massive atelectasis of the left lung with signs of intraparenchymal air trapping.

In the following weeks the clinical course was characterized by recurrent respiratory upper and lower airways infections, that required monthly treatment with systemic antibiotics, and progressive dyspnoea and tachypnoea. At 9 months a chest high resolution computed tomography (HRCT) performed because of lower respiratory tract infection unresponsive to prolonged antibiotics therapy, revealed massive atelectasis of the left lung with diffuse air-trapping (Figure 2). A significant increase in heart volume was also evident, and this finding appeared consistent with a possible compressive effect of the massive cardiomegaly on the left main bronchus. In the following weeks the SaO_2_ at room air progressively decreased, and O_2_ supplementation, combined with a daily program of chest physiotherapy, was started. At the age of 10 months, after a further episode of pneumonia, the patient developed acute respiratory failure with severe hypoxemia and hypercapnia (PaO_2_ 36 mmHg, PaCO_2_ 68 mmHg) that required the admission to the Paediatric Intensive Care Unit. Despite mechanical ventilation, her condition progressively worsened, and she eventually died. The parents did not authorise an autopsy.

## Discussion

The most severe form of Pompe disease is typically diagnosed in infants between 3 and 5 months of age during the assessment of a respiratory infection, cardiomegaly, or hypotonia [2]. However, symptoms and signs of the disease might be neglected or underestimated, this resulting in delayed referral to specialized centers for diagnostic confirmation. In the described case, the hospital admission was required because of suspected liver disease, and the clinical picture of generalized hypotonia was not reported as the main clinical problem of the baby.

Severe hypotonia of the skeletal muscles associated with breathing (diaphragm, intercostal and accessory muscles) is described in Pompe disease, this resulting in inability to generate normal levels of pressure and flow in the airways during inspiration and expiration [7-9]. These events significantly impair the removal of secretions from the airways, predisposing to even severe recurrent respiratory infections that affect morbidity and mortality of patients [2]. Additional mechanisms, such as the aspiration of infected secretions and/or of food in the airways, can eventually result in the development of lung lesions including segmental to lobar atelectasis and bronchiectasis. A recent study of 13 children with Pompe disease highlighted the central role of videofluoroscopy in detecting swallowing disturbances that were evident in most of the study population [10]. In our patient, the association of massive cardiomegaly with respiratory muscle weakness likely explains the development of progressive lung damage and irreversible respiratory failure.

During the past two decades, impressive progress has been made in the treatment of Pompe disease [11]. Indeed, despite enzyme replacement therapy is currently the only clinically approved treatment, different therapeutic approaches are under investigation, including enzyme enhancement therapy by pharmacological chaperones and gene therapy [1,11,12]. Furthermore, substrate reduction therapy has been proved to significantly improve structural, metabolic and functional defects in a murine model of glycogenosis type II, offering a new perspective for the future treatment of this condition [13].

Rarity of the disease and variations in clinical presentation often result in considerable delay in the diagnosis of Pompe disease, and a severely impaired health status already at time of diagnosis has been reported [14]. These observations, associated with the better clinical outcomes achieved when enzyme replacement therapy is started early, highlight the importance of timely detection of Pompe disease [14]. Particularly, since methods for dried bloodspot screening are currently being explored, neonatal screening represents the most promising option to enable earlier diagnosis, and a recent study has shown that nationwide testing of GAA-activity in dried bloodspots substantially reduced the diagnostic delay for infants with classic infantile Pompe disease [15]. Nevertheless, the impossibility of currently available screening techniques to discriminate between classic infantile Pompe disease and less progressive variants of the condition raises a number of ethical, legal and social considerations that warrant further research to explore the potential of neonatal screening of this disease.

In patients with Pompe disease not treated progressive respiratory failure is an important cause of morbidity and mortality [2]. The age at death depends on the speed of progression of the symptoms and the extent of muscle involvement [2]. In natural history studies of infants, the gap between diagnosis and ventilator use or death was 1 to 2 months, and nearly all cases died by the age of 18 months [16].

Unfortunately, enzyme replacement therapy has been available in Italy only from 2006, and at the time the patient was assisted at our Department (2002) we could not prescribe it. Despite not all patients benefit equally from this therapeutic approach and, particularly, cross-reactive immunologic material-negative subjects have showed poor clinical response [17], favorable outcomes with early intravenous enzyme replacement therapy have been reported [16,18-21]. In particular, treated patients have lower risk of undergoing mechanical ventilation, with a 100% survival rate at 18-month [18]. Finally, the benefits of long term therapy appear evident even at the age of 36 months [19].

## Conclusions

Respiratory manifestations seriously complicate the course of Pompe disease through several mechanisms (generalized muscle hypotonia, aspiration pneumonia, development of pulmonary atelectasis) and are often the cause of premature death. Early diagnosis is crucial to improve the final outcome of the disorder. Therefore, recognition of even subtle symptoms and signs is strongly recommended since early treatment significantly improves patients’ survival and may definitely change the natural history of the disorder. Finally, since glycogen storage disorders are multisystemic, the individual management should be handled by a multidisciplinary team of specialists including pediatricians expert in inborn errors of metabolism, pulmonologists, cardiologists, radiologists and respiratory therapists.

### Consent

Written informed consent was obtained from the patient’s guardian/parent/next in keen for publication of this report and any accompanying images. A copy of the written consent is available for review by the Editor-in-Chief of this journal.

## Abbreviations

GAA: Acid α-glucosidase; CK: Creatine kinase; AST: Asparate aminotransferase; LDH: Lactate dehydrogenase; SaO_2_: Oxygen saturation; ALT: Alanine aminotranspherase; ALP: Alkaline phosphatase; HRCT: High resolution computed tomography.

## Competing interests

The authors declare that they have no competing interests.

## Authors’ contributions

FS has made substantial contributions to conception and design, has been involved in drafting the manuscript, and has given final approval of the version to be published. SDS has made substantial contributions to conception and design, has been involved in drafting the manuscript, and has given final approval of the version to be published. SM has made substantial contributions to acquisition of data, has been involved in drafting the manuscript, and has given final approval of the version to be published. MM has made substantial contributions to acquisition of data has been involved in drafting the manuscript, and has given final approval of the version to be published. RDC has made substantial contributions to acquisition of data, has been involved in revising the manuscript critically for important intellectual content, and has given final approval of the version to be published. EA has made substantial contributions to conception and design, has been involved in revising the manuscript critically for important intellectual content, and has given final approval of the version to be published. CP has made substantial contributions to conception and design and analysis and interpretation of data, has been involved in revising the manuscript critically for important intellectual content, and has given final approval of the version to be published. MS has made substantial contributions to analysis and interpretation of data, has been involved in revising the manuscript critically for important intellectual content, and has given final approval of the version to be published. GP has made substantial contributions to conception and design and analysis and interpretation of data, has been involved in drafting the manuscript and revising it critically for important intellectual content, and has given final approval of the version to be published. All authors read and approved the final manuscript.
